# Characterization of a novel rabbit model of Peyronie’s disease

**DOI:** 10.1038/s41443-023-00671-y

**Published:** 2023-02-13

**Authors:** Gokhan Gundogdu, Travis Nguyen, Aarthi Namasivayam, Stephanie Starek, Joel Gelman, Joshua R. Mauney

**Affiliations:** 1 Department of Urology, University of California, Irvine, Orange, CA USA; 2 Department of Biomedical Engineering, University of California, Irvine, Irvine, CA USA

**Keywords:** Sexual dysfunction, Biotechnology

## Abstract

Peyronie’s disease (PD) is a debilitating pathology which is associated with penile curvature and erectile dysfunction due to the formation of fibrotic plaques in the penile tunica albuginea. In the present study, we developed a novel rabbit model of PD via subtunical injection of recombinant transforming growth factor (TGF)-β1 protein and characterized erectile function and histopathological endpoints following plaque formation. Ten adult male, New Zealand white rabbits were randomized into 3 experimental groups including nonsurgical controls (NSC, *N* = 3) and those receiving subtunical injections of vehicle (*N* = 3) or TGF-β1 protein (0.5 µg/50 µl; *N* = 4). Following 1 month post-op, focal fibrous plaques composed of disorganized collagen type I and III bundles as well as fragmented elastin fibers at TGF-β1 injection sites were observed in contrast to control groups. Cavernosometric and cavernosographic evaluations revealed no significant differences in maximum intracorporal pressures or substantial curvature during papaverine-induced erection in either the vehicle or TGF-β1 cohorts. Immunohistochemical and histomorphometric analyses demonstrated significant increases in elastase 2B expression in TGF-β1-induced plaques as well as significant declines in matrix metalloproteinase (MMP)-2 and -9 expression relative to control levels. Our results demonstrate that PD-like fibrotic plaques can be created in the rabbit penile tunica albuginea following TGF-β1 injection.

## Introduction

Peyronie’s disease (PD) is a benign connective tissue pathology that affects between 0.4% and 13% of the male population [1]. This condition can result in penile curvature and subsequent erectile dysfunction due to localized formation of fibrotic plaques in the penile tunica albuginea surrounding the corpus cavernosum [2]. The etiology of PD is poorly understood, however inflammation of the tunica albuginea in response to recurrent sexual trauma has been implicated as a putative driver of scar tissue formation and subsequent plaque development [3]. In addition, PD fibrogenesis is also exacerbated by imbalances between extracellular matrix (ECM) proteolytic enzymes including matrix metalloproteinases (MMP) and tissue inhibitors of metalloproteinases (TIMP) which led to abnormal degradation and accumulation of ECM components within the plaque microenvironment [4]. Overall, PD is a debilitating disease which significantly impacts patient quality of life due to declines in sexual activity from pain and penile deformity as well as adverse psychological effects from disruptions in partner intimacy [5].

Preclinical animal models of PD represent valuable tools for studying the pathophysiology of disease progression as well as provide an in vivo setting for evaluating prospective therapeutic interventions prior to human translation. Current animal models available for PD investigations are predominantly based in rats [6]. These systems involve induction of focal inflammatory events in the tunica albuginea via injection of pro-fibrotic agents such as transforming growth factor-β1 (TGF-β1), cytomodulin, fibrin or myostatin as well as surgical trauma to elicit myofibroblast differentiation and subsequent abnormal collagen deposition and elastin fiber degradation culminating in plaque development [7–10]. Unfortunately, these rodent models are limited by inconsistencies in the timing and duration of plaque formation and do not recapitulate severe penile curvature or plaque calcification observed in humans [6]. Moreover, the small size of the rat penis precludes corporoplasty studies needed to evaluate the performance of novel surgical grafts for resolution of chronic PD following plaque excision. In the present report, our goal was to create a new PD model in male rabbits via subtunical injection of recombinant TGF-β1 protein and characterize papaverine-induced, erectile function and histopathological outcomes following plaque formation. Rabbits have several advantages as a model organism for PD studies over rats. For instance, rabbits are commonly used for penile and urethral reconstructive studies [11, 12] and the size of the penis allows for targeted tunica albuginea injections of fibrotic cytokines with minimal risk of extravasation into the penile corpora. In addition, male rabbits lack a baculum [13], similar to humans and in contrast to rats [14], thus allowing for assessments of penile curvature during PD progression without mechanical resistance from rigid osseous structures.

## Materials and methods

Ten adult male, New Zealand white rabbits (Western Oregon Rabbit Co. Philomath, OR USA) weighing 3.5–4 kg were randomized into three experimental cohorts including nonsurgical controls (NSC, *N* = 3) and those receiving subtunical injections of vehicle (*N* = 3) consisting of 4 mM hydrochloric acid and 1 mg/ml bovine serum albumin or recombinant human TGF-β1 protein (*N* = 4). Sample sizes for each group were determined based on our past reported experiments which utilized similar animal replicates per group to determine significance in quantitative assessments [12]. Rabbits were single housed postoperatively, fed with regular rabbit food and allowed free access to water throughout the study. The Institutional Animal Care and Use Committee (IACUC) of University of California, Irvine approved the study protocol (# AUP-20-077) and all experimental procedures adhered to the National Institutes of Health’s Guidelines for the Care and Use of Laboratory Animals. This study was also conducted in compliance with ARRIVE guidelines (https://arriveguidelines.org).

### Surgical procedures

**Fig. 1:**
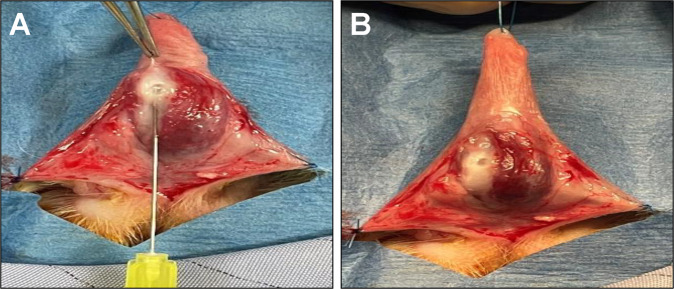
Rabbit Peyronie’s disease model. Overview of surgical stages of model creation. **A** Penile skin and Buck’s fascia were incised longitudinally in male rabbits (*N* = 7). A 30-gauge needle was slightly inserted into the penile tunica albuginea between the urethra and right lateral vascular structures and vehicle and TGF-β1 injections were performed. **B** Prominent white discoloration with central bullae were observed at injection sites of all study animals.

Anesthesia was induced by subcutaneous injection of 35 mg/kg Ketamine (Zoetis Inc, Kalamazoo, MI, USA) and 5 mg/kg Xylazine (MWI, Boise, ID, USA) and maintained by isoflurane via mask inhalation. Animals were placed in a supine position and excess fur was trimmed around the genital area. The surgical field was scrubbed with povidone-iodine solution and 70% ethanol three times and sterilely draped. A 5-0 polyprolene (Ethicon, Somerville, NJ, USA) stay suture was positioned at the tip of the distal penile skin to facilitate surgical manipulations. The skin web located between the penis and anus was divided and a ventral incision was made longitudinally between the penile skin and Buck’s fascia. Subtunical injection (50 µl) of vehicle or TGF-β1 protein (0.5 µg, R&D Systems, Minneapolis, MN) was performed with a 30-gauge needle at a single location in the tunica albuginea ~1.5 cm above the penile radix and between the urethra and lateral neurovascular bundle (Fig. 1). Absorbable 5-0 polyglactin sutures (Covidien, Mansfield, MA, USA) were then utilized to close skin incisions. Rabbits were dressed with Elizabethan collars for 5 days to avoid self-mutilation of the surgical site. For 7 days post-op, animals were evaluated daily to assess any potential surgical complications and then monitored weekly thereafter until scheduled euthanasia at 1 month. Pain management was facilitated by a single subcutaneous injection of Buprenorphine SR (0.12 mg/kg, ZooPharm, Laramie, WY, USA) immediately after the surgery as well as daily subcutaneous injections of Banamine (1 mg/kg, Merck Animal Health, Kenilworth, NJ, USA) for 3 days. Rabbits were also given subcutaneous injection of Enrofloxacin (5 mg/kg, Baytril®100; Bayer Healthcare LLC, KA, United States) prior to surgery and for 3 days postoperatively to prevent bacterial infection.

### Cavernosography and cavernosometry

Cavernosographic and cavernosometric evaluations were performed 1 month following subtunical injections in experimental animals to assess papaverine-induced, erectile function using previously reported protocols [12, 15]. Rabbits were supine positioned under general anesthesia described above and a stay suture was placed at the tip of glans. The penile skin was degloved and a 22-gauge IV catheter was inserted into the right cavernous body below the subtunical injection site at the level of penile radix. For cavernosography, contrast medium (Omnipaque 300; GE Healthcare Inc., Marlborough, MA, USA) diluted with 1:1 saline was infused into the cavernous body through the IV catheter. Serial X-ray images were acquired in the anterior/posterior and lateral directions by using a C-arm fluoroscope (BV Pulsera; Philips, Eindhoven, Netherlands) while the penis was at its maximal erection point during contrast infusion. For cavernosometry, a second 22-gauge IV catheter was inserted into the left cavernous body adjacent to the penile radix. A urodynamics system (Goby CT; Laborie, ON, Canada) was connected to the right catheter for continuous recording of intracorporal pressures (ICP). Baseline ICP levels were recorded and then heparinized saline (10 U/ml) was infused (1 ml/min) into the corporal bodies in combination with the vasodilator, Papaverine-HCl (15 mg/ml, Sigma-Aldrich, Inc; MO, USA) to induce penile erection. Maximum ICP values were acquired and maintained for a period of 10 min with saline infusion. Photomicrographs of penile erections were captured at maximum ICP levels. Rabbits in both experimental groups as well as NSC were euthanized by intravenous injection of 0.2 ml/kg pentobarbital sodium and phenytoin sodium euthanasia solution (Euthasol; Virbac AH, Westlake, TX, USA) and tissues were collected for histological, immunohistochemical (IHC), and histomorphometric analyses.

### Histological, immunohistochemical, and histomorphometric analyses

Penile tissues harvested from NSC as well as vehicle and TGF-β1-treated cohorts following 1 month post-op were excised for routine histological procedures following animal harvest. Tissue specimens were fixed in 10% neutral-buffered formalin for 12 h, dehydrated in graded alcohols, and paraffin embedded using standard protocols. Five micron sections were stained with Masson’s trichrome (MTS) and picrosirius red (PSR) to visualize total collagen content as previously described [16, 17]. Parallel sections in all groups were also evaluated for the presence of elastin fibers in control tissues and PD plaques using a commercially available, Verhoeff Van Gieson (VVG) staining kit (ab150667, Abcam, Cambridge, MA, USA). IHC evaluations were performed on tissue sections following antigen retrieval (10 mM sodium citrate buffer, pH 6.0) and incubation in phosphate-buffered saline with 0.3% Triton X-100, 5% fetal bovine serum, and 1% bovine serum albumin for 1 h at room temperature. Sections were then independently stained with primary antibodies including anti-collagen type I (ab34710, 1:200 dilution, Abcam), anti-collagen type III (ab7778, 1:50 dilution, Abcam), anti-MMP1 (10371-2-AP, 1:200 dilution, Proteintech, Rosemont, IL, USA), anti-MMP2 (436000, 1:150 dilution, Invitrogen, Waltham, MA), anti-MMP9 (AV33090, 1:200 dilution, Sigma-Aldrich), anti-MMP12 (22989-1-AP, 1:100 dilution, Proteintech), anti-TIMP1 (BS-0415R, 1:200 dilution, Bioss, Woburn, MA, USA), and anti-elastase 2B (orb183368, 1:100 dilution, Biorbyt, St Louis, MO, USA). Samples were then incubated with species-matched, horseradish peroxidase (HRP)-conjugated secondary antibodies and 3,3’Diaminobenzidine (DAB) substrate followed by hematoxylin counterstain. Sample visualization was performed with a Zeiss Axio Imager M2 model (Carl Zeiss MicroImaging, Thornwood, NY) and representative fields were captured with Zen software (version 3.1). Parallel specimens stained with secondary antibodies alone served as negative controls and led to no detectable background signal. For histomorphometric evaluations, quantitation of selective marker expression was calculated across 2–3 independent global sections per group replicate and displayed as a proportion of stained area per total field area examined utilizing ImageJ software. Analyses were performed with nonblinded observers.

### Statistics

Statistical analyses of quantitative data between groups was performed using the Mann–Whitney *U* test considering a value of *p* ≤ 0.05 as significant. Quantitative data were represented as mean ± standard deviation (SD).

## Results

**Fig. 2:**
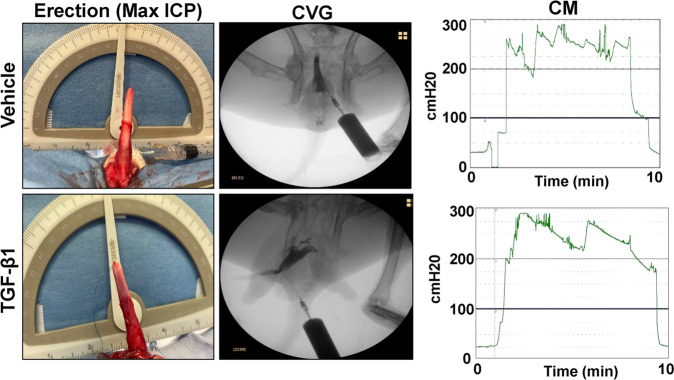
Cavernosometric and cavernosographic assessments of erectile function. Representative photomicrographs of penile erections (first column) and cavernosographic (CVG) images (second column) at maximum intracorporal pressure (ICP) levels following contrast instillation in vehicle and TGF-β1-treated rabbits following 1 month post-op. Representative intracorporal pressure (ICP) tracings from animals described above subjected to cavernosometric analysis (CM, third column). Data are representative of *N* = 3–4 animals for each analysis.

There were no intraoperative or immediate postoperative complications encountered in any rabbits following subtunical injections except transient mild edema and swelling in the original surgical area. Papaverine-induced, penile erectile function was assessed by cavernosography and cavernosometry in vehicle and TGF-β1-treated animals at 1 month post-op (Fig. 2). Following injection of contrast agent, the corpora cavernosa in each group filled with fluid in a homogenous pattern resulting in an erection in the absence of leakage, filling defects or substantial curvature. Cavernosometric evaluations were performed to ascertain the maximum vascular pressure in the corpus cavernosum following papaverine-induced erection. Full erections were achieved and sustained for 10 min in all animals. No significant differences in maximum ICP values were observed between vehicle (275 ± 23 cmH_2_O) and TGF-β1-treated rabbits (291 ± 14 cmH_2_O) (Mann–Whitney *U* test, *p* = 0.211). These results demonstrate that subtunical TGF-β1 injections did not lead to deficiencies in papaverine-induced, erectile function during the study period.

**Fig. 3:**
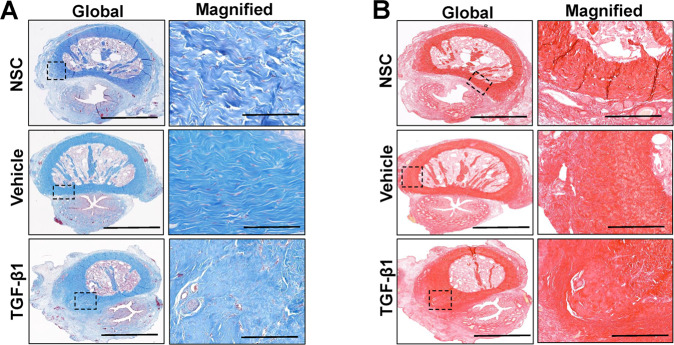
Histological evaluations of plaque formation. Representative photomicrographs of global and magnified rabbit penile cross-sections stained with **A** Masson’s trichrome (MTS) or **B** Picrosirius red (PSR) in nonsurgical controls (NSC) as well as vehicle and TGF-β1-treated groups at 1 month post-op. Boxed areas denote magnified native and vehicle-treated tunica albuginea or fibrotic plaques induced by TGF-β1 injection. Scale bars for photomicrographs in global and magnified panels are 3 mm and 400 µm, respectively. Data are representative of *N* = 3–4 animals per group.

**Fig. 4:**
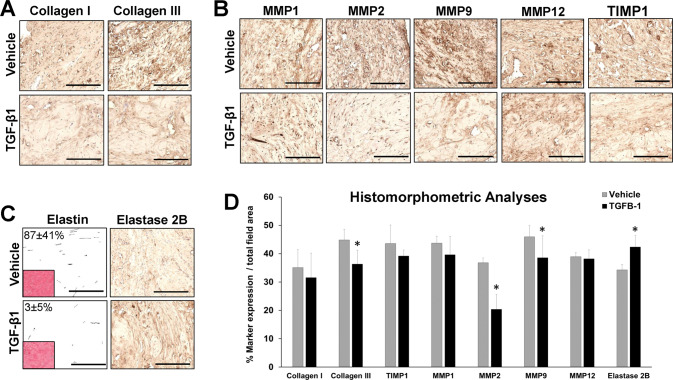
Immunohistochemical and histomorphometric analyses of ECM components and proteolytic enzymes in vehicle and TGF-β1-treated specimens. Representative photomicrographs of collagen I and III expression (**A**) as well as associated proteolytic enzymes (MMP) and their inhibitors (TIMP) (**B**) in the penile tunica albuginea of vehicle-treated samples and TGF-β1-induced plaques. Marker expression is denoted in brown (HRP labeling). **C** Relative elastin fiber density (VVG stain, inset; grayscale converted fibers) and elastase 2B expression (HRP labeling) in samples described in **A**, **B**. Scale bars for all panels in **A**–**C** are 400 µm. **D** Quantitative assessments of marker expression described in (**A**–**C**). *p ≤ 0.05 in comparison to respective vehicle controls. *N* = 3–4 samples per data point. Results from all groups were analyzed with the Mann–Whitney *U* test. Values displayed as means ± SD.

Histological (MTS, PSR, VVG), IHC and histomorphometric evaluations of the tunica albuginea in penile cross-sections from NSC as well as vehicle and TGF-β1-treated rabbits were performed to characterize levels of ECM components (collagens I and III, elastin fibers), proteolytic enzymes (MMP1, 2, 9, 12, and elastase 2B) and associated inhibitors (TIMP1). MTS (Fig. 3A) and PSR (Fig. 3B) analyses revealed that all rabbits treated with TGF-β1 protein displayed the formation of a focal fibrous plaque at the subtunical injection site which was composed of disorganized collagen fibrils. IHC assessments (Fig. 4) demonstrated that fibrous plaques in the TGF-β1 group contained collagens type I and III with relative expression levels which were respectively 91% (Mann–Whitney *U* test, *p* = 0.378) and 80% (Mann–Whitney *U* test, *p* = 0.00030) of vehicle controls. In addition, quantitation of MMP/TIMP expression levels in TGF-β1 induced plaques revealed significant reductions in MMP2 and MMP9 proteins corresponding to 54% (Mann–Whitney *U* test, *p* = 0.00000069) and 85% (Mann–Whitney *U* test, *p* = 0.0058) of vehicle controls. However, the expression patterns of MMP1, MMP12, and TIMP1 proteins were not significantly different between the cohorts (Mann–Whitney *U* test: MMP1, *p* = 0.204; MMP12, *p* = 0.769; TIMP1, *p* = 0.097). Elastin degradation (VVG) within the fibrous plaques was evident following TGF-β1 treatment with relative elastin fiber density observed at ~3% of vehicle controls (Mann–Whitney *U* test, *p* = 0.05). Elastase 2B protein expression was also significantly elevated in fibrous plaques to levels 124% relative to the vehicle group (Mann–Whitney *U* test, *p* = 0.0021). These data highlight the changes in ECM composition as well as proteolytic enzyme and inhibitor expression which occur during plaque formation following subtunical TGF-β1 injection in rabbits.

## Discussion

The goal of this study was to create a novel rabbit model of PD and to characterize the functional and histological changes which occur in the penis during fibrotic remodeling of the tunica albuginea in response to TGF-β1 treatment. The choice to utilize TGF-β1 protein as a disease inducer was based on previous observations demonstrating this pro-fibrotic cytokine is overexpressed in human PD lesions [18]. In addition, overactivation of TGF-β1 signaling seems to be a pathogenetic factor in the development of PD since the expression and activity of TGF-β1-induced Smad transcription factors is increased in fibroblasts of patients with PD [19]. Moreover, subtunical injection of TGF-β1 protein also leads to the formation of PD plaques in rats [6, 20], while regression of these lesions can be achieved through inhibition of TGF-β1 type I receptor activation [21, 22]. In response to repeated sexual trauma, TGF-β1 signaling in combination with oxidative stress [23] and platelet and coagulation pathway activation [2] serve as major regulators of fibrotic and inflammatory processes governing plaque development in PD [24].

Our results demonstrated the formation of focal fibrous plaques composed of disorganized collagen type I and III bundles as well as fragmented elastin fibers at TGF-β1 injection sites following 1 month post-op. These histopathological features were similar to those previously observed in human PD plaques which display significant elastin fiber degradation as well as fenestration and disorganization of collagen bundles [18, 25]. However, there was no detectable plaque calcification or apparent penile curvature in TGF-β1-treated specimens as previously observed in patients with chronic PD [26] suggesting our model system leads to acute disease progression. Past studies have demonstrated that repeated subtunical injection of adenovirus expressing TGF-β1 in rodents results in features reminiscent of chronic PD including severe penile deformities and calcified plaques [27]. Therefore, future experiments will investigate the ability of recurrent subtunical injections of TGF-β1 protein in rabbits to induce a chronic PD phenotype.

Erectile dysfunction secondary to PD in humans is often a consequence of plaque-associated, penile structural alterations and/or corporal veno-occlusive abnormalities which lead to disruptions in normal penile hemodynamics during sexual activity [28, 29]. Evaluation of papaverine-induced, penile erectile function in TGF-β1-treated rabbits revealed no filling defects or significant differences in maximum ICP values in comparison to vehicle controls. These results are consistent with a lack of corporal fibrosis and severe penile angulation in the TGF-β1-treated cohort. In rat models of PD, TGF-β1-induced fibrotic plaques extend into cavernosal tissues and mitigate peak ICP values during erectile stimulation via excessive ECM deposition and loss of smooth muscle cells in the corpus cavernosa [30]. However, this situation is markedly different in humans afflicted with PD wherein corporal fibrosis is rare and plaques are normally sequestered in the tunica albuginea [31]. Due to the relatively small size of the rat penis, corporal fibrosis in rat PD models is likely due to imprecise injection of TGF-β1 into the subtunical space which then permeates into the corpus cavernosa activating myofibroblasts and aberrant ECM formation. In contrast, the larger size of the rabbit penis in our PD model allows for more accurate targeting of the tunica albuginea for pathological insult without compromising the integrity of corpus cavernosa. Therefore, our rabbit model has the potential advantage of decoupling corporal fibrosis from plaque development allowing for direct assessments of the impact of PD on erectile function. Moreover, the dimensions of the rabbit phallus also allow for corporoplasty procedures to be performed with autologous tissue grafts or tissue engineered constructs [12] to vet prospective repair strategies for PD patients who fail to respond to minimally invasive treatment approaches. This feature highlights another potential attribute of using rabbits to develop a PD model in comparison to rats wherein the small size of the penis precludes therapeutic graft screening in the latter.

Plaque fibrosis in PD is a consequence of dysregulation of ECM proteolytic enzymes and their inhibitors which leads to aberrant ECM production and subsequent scar tissue formation. Previous profiling studies of human PD plaques has revealed increases in collagen types I and III with concurrent reductions in elastin fiber density relative to healthy tissues [32–35]. The putative cause for excessive collagen accumulation in PD patients has been linked to alterations in the balance between MMP/TIMP activities in the plaque microenvironment wherein increases in TIMP expression occur in the absence of perturbations of MMP family members thus favoring collagen stability [4, 35]. In our model, no significant increases in collagen types I and III were detected in TGF-β1-induced plaques relative to controls despite significant reductions in MMP2 and MMP9 expression in the diseased cohort. The absence of significant increases in TIMP1 expression in TGF-β1-treated rabbits over control levels may explain the lack of collagen accumulation in these specimens. The discrepancy in collagen and MMP/TIMP variations observed in human and rabbit PD lesions may reflect different states of disease progression since analysis of clinical specimens is often performed on patients with advanced disease following corporoplasty [4, 35]. In contrast, our data did reflect a significant upregulation of elastase 2B in the TGF-β1 cohort in comparison to controls which may explain the reductions in elastin fiber density in this group. Indeed, enrichment of elastase 2B has been previously identified in gene expression screens of human PD plaques [36] and degradation and disorganization of elastin fiber networks are known to contribute to scar tissue development in skin diseases [37]. Taken together, activation of elastase 2B and subsequent degradation of elastin fibers in the tunica albuginea may play a more pronounced role in acute phases of PD plaque formation than MMP/TIMP dysregulation.

There were several key limitations in our experimental design which will be addressed in future studies. Although, papaverine-induced, cavernosometric and cavernosometric assessments revealed no detectable penile anomalies following TGF-β1 treatment, our assay methodology was not physiological in nature. Therefore, validation of in vivo erectile function with ICP normalized to mean arterial pressure following electrical stimulation of the cavernosal nerve is needed for further characterization [38]. In addition, one month following TGF-β1 injection, endpoint analyses revealed our rabbit PD model primarily mimicked acute stages of plaque formation without evidence of tunica albuginea calcification or serve angulation seen in chronic pathologies. Improvements in our rabbit PD model will focus on delineating experimental conditions such as frequency, dose, and duration of subtunical fibrotic insults which can promote further disease development into severe phenotypes for potential therapeutic testing.

In summary, we have established a new model of PD in rabbits wherein subtunical injection of TGF-β1 protein leads to the formation of fibrotic plaques reminiscent of acute stages of human pathology. Our results demonstrate that elevations in elastase 2B expression and elastin fiber degradation are significantly associated with initial plaque development and may represent potential therapeutic targets for disease intervention. Future investigations will focus on the efficacy of elastase inhibitors to mitigate TGF-β1-induced plaques in rabbits.

## Data Availability

The experimental data generated for this study are available on request to the corresponding author.

## References

[1] DiBenedetti DB, Nguyen D, Zografos L, Ziemiecki R, Zhou X (2011). A population-based study of Peyronie’s disease: prevalence and treatment patterns in the United States. Adv Urol.

[2] Gonzalez-Cadavid NF, Rajfer J (2005). Mechanisms of disease: new insights into the cellular and molecular pathology of Peyronie’s disease. Nat Rev Urol.

[3] Jarow JP, Lowe FC (1997). Penile trauma: an etiologic factor in Peyronie’s disease and erectile dysfunction. J Urol.

[4] Watanabe MS, Theodoro TR, Coelho NL, Mendes A, Leonel MLP, Mader AM (2017). Extracellular matrix alterations in the Peyronie’s disease. J Adv Res.

[5] Goldstein I, Hartzell R, Shabsigh R (2016). The impact of peyronie’s disease on the patient: gaps in our current understanding. J Sex Marital Ther.

[6] Chung E, De Young L, Brock GB (2011). Rat as an animal model for Peyronie’s disease research: a review of current methods and the peer-reviewed literature. Int J Impot Res.

[7] El-Sakka AI, Hassoba HM, Chui RM, Bhatnagar RS, Dahiya R, Lue TF (1997). An animal model of Peyronie’s-like condition associated with an increase of transforming growth factor beta mRNA and protein expression. J Urol.

[8] Bivalacqua TJ, Diner EK, Novak TE, Vohra Y, Sikka SC, Champion HC (2000). A rat model of Peyronie’s disease associated with a decrease in erectile activity and an increase in inducible nitric-oxide synthase protein expression. J Urol.

[9] Davila HH, Ferrini MG, Rajfer J, Gonzalez-Cadavid NF (2003). Fibrin as an inducer of fibrosis in the tunica albuginea of rat: a new animal model of Peyronie’s disease. BJU Int.

[10] Cantini LP, Ferrini MG, Vernet D, Magee TR, Qian A, Gelfand RA (2008). Profibrotic role of myostatin in Peyronie’s disease. J Sex Med.

[11] Algarrahi K, Affas S, Sack BS, Yang X, Costa K, Seager C (2018). Repair of injured urethras with silk fibroin scaffolds in a rabbit model of onlay urethroplasty. J Surg Res.

[12] Gundogdu G, Okhunov Z, Starek S, Veneri F, Orabi H, Holzman SA (2021). Evaluation of Bi-layer silk fibroin grafts for penile tunica albuginea repair in a rabbit corporoplasty model. Front Bioeng Biotechnol.

[13] Skonieczna J, Madej JP, Kaczmarek-Pawelska A, Będziński R (2021). Histological and morphometric evaluation of the urethra and penis in male New Zealand White rabbits. Anat Histol Embryol.

[14] Goyal HO, Braden TD, Williams CS, Dalvi P, Mansour MM, Mansour M (2004). Abnormal morphology of the penis in male rats exposed neonatally to diethylstilbestrol is associated with altered profile of estrogen receptor-alpha protein, but not of androgen receptor protein: a developmental and immunocytochemical study. Biol Reprod.

[15] Chen KL, Eberli D, Yoo JJ, Atala A (2010). Bioengineered corporal tissue for structural and functional restoration of the penis. Proc Natl Acad Sci USA.

[16] Junqueira LC, Bignolas G, Brentani RR (1979). Picrosirius staining plus polarization microscopy, a specific method for collagen detection in tissue sections. Histochem J.

[17] Davis CJ (1997). The microscopic pathology of Peyronie’s disease. J Urol.

[18] El-Sakka AI, Hassoba HM, Pillarisetty RJ, Dahiya R, Lue TF (1997). Peyronie’s disease is associated with an increase in transforming growth factor-beta protein expression. J Urol.

[19] Haag SM, Hauck EW, Szardening-Kirchner C, Diemer T, Cha ES, Weidner W (2007). Alterations in the transforming growth factor (TGF)-beta pathway as a potential factor in the pathogenesis of Peyronie’s disease. Eur Urol.

[20] Gokce A, Abd Elmageed ZY, Lasker GF, Bouljihad M, Kim H, Trost LW (2014). Adipose tissue-derived stem cell therapy for prevention and treatment of erectile dysfunction in a rat model of Peyronie’s disease. Andrology.

[21] Piao S, Choi MJ, Tumurbaatar M, Kim WJ, Jin HR, Shin SH (2010). Transforming growth factor (TGF)-β type I receptor kinase (ALK5) inhibitor alleviates profibrotic TGF-β1 responses in fibroblasts derived from Peyronie’s plaque. J Sex Med.

[22] Ryu JK, Piao S, Shin HY, Choi MJ, Zhang LW, Jin HR (2009). IN-1130, a novel transforming growth factor-beta type I receptor kinase (activin receptor-like kinase 5) inhibitor, promotes regression of fibrotic plaque and corrects penile curvature in a rat model of Peyronie’s disease. J Sex Med.

[23] Sikka SC, Hellstrom WJ (2002). Role of oxidative stress and antioxidants in Peyronie’s disease. Int J Impot Res.

[24] Zhang F, Qin F, Yuan J (2021). Molecular mechanisms and current pharmacotherapy of Peyronie’s disease: a review. Front Pharmacol.

[25] Moreland RB, Nehra A (2002). Pathophysiology of Peyronie’s disease. Int J Impot Res.

[26] Wymer K, Ziegelmann M, Savage J, Kohler T, Trost L (2018). Plaque calcification: an important predictor of collagenase clostridium histolyticum treatment outcomes for men with Peyronie’s disease. Urology..

[27] Piao S, Ryu JK, Shin HY, Zhang L, Song SU, Han JY (2008). Repeated intratunical injection of adenovirus expressing transforming growth factor-beta1 in a rat induces penile curvature with tunical fibrotic plaque: a useful model for the study of Peyronie’s disease. Int J Androl.

[28] Gasior BL, Levine FJ, Howannesian A, Krane RJ, Goldstein I (1990). Plaque-associated corporal veno-occlusive dysfunction in idiopathic Peyronie’s disease: a pharmacocavernosometric and pharmacocavernosographic study. World J Urol.

[29] Levine LA, Latchamsetty KC (2002). Treatment of erectile dysfunction in patients with Peyronie’s disease using sildenafil citrate. Int J Impot Res.

[30] Li J, Wang S, Qin F, Zhu M, You X, Wu C (2018). Reduction in Peyronie’s-like plaque size using a vacuum erection device in a rat model of Peyronie’s disease via the TGF-β/SMAD signaling pathway. Andrologia..

[31] Ro JY, Divatia MK, Kim KR, Amin MB, Ayala AG. Penis and scrotum. In: Cheng L, MacLennan GT, Bostwick DG, editors. Urologic surgical pathology. 4th ed. Philadelphia: Elsevier; 2020. p. 853–901.

[32] Chiang PH, Chiang CP, Shen MR, Huang CH, Wang CJ, Huang IY (1992). Study of the changes in collagen of the tunica albuginea in venogenic impotence and Peyronie’s disease. Eur Urol.

[33] Brock G, Hsu GL, Nunes L, von Heyden B, Lue TF (1997). The anatomy of the tunica albuginea in the normal penis and Peyronie’s disease. J Urol.

[34] Ten Dam EPM, van Driel MF, de Jong IJ, Werker PMN, Bank RA (2020). Glimpses into the molecular pathogenesis of Peyronie’s disease. Aging Male.

[35] Del Carlo M, Cole AA, Levine LA (2008). Differential calcium independent regulation of matrix metalloproteinases and tissue inhibitors of matrix metalloproteinases by interleukin-1beta and transforming growth factor-beta in Peyronie’s plaque fibroblasts. J Urol.

[36] Magee TR, Qian A, Rajfer J, Sander FC, Levine LA, Gonzalez-Cadavid NF (2002). Gene expression profiles in the Peyronie’s disease plaque. Urology..

[37] Baumann L, Bernstein EF, Weiss AS, Bates D, Humphrey S, Silberberg M (2021). Clinical relevance of elastin in the structure and function of skin. Aesthet Surg J Open Forum.

[38] Abdulmaged M, Park TK, Dhir V, Kim NN, Moreland R, Goldstein B (1999). Effects of castration and androgen replacement on erectile function in a rabbit model. Endocrinology..

